# Association of genotype of *POU1F1* intron 1 with carcass characteristics in crossbred pigs

**DOI:** 10.1186/2055-0391-56-25

**Published:** 2014-11-27

**Authors:** Gye-Woong Kim, Jae-Young Yoo, Hack-Youn Kim

**Affiliations:** Department of Animal Resources, Kong-Ju National University, # 54 Daehakro, Yesan, Chungnam, 340-702 Korea; Department of Obstetrics and Gynecology, Ewha Woman’s University, Seoul, 158-710 Korea

**Keywords:** *POU1F1*, Carcass characteristics, Genotypes, Gene, Intron 1

## Abstract

This study was carried out to investigate the association of *POU1F1* (POU domain, class 1, transcription factor 1, Pit1, renamed as *POU1F1*) gene with backfat thickness (mm), carcass weight (kg), pH, and color values (L^*^, a^*^, b^*^) in crossbred pigs (Landrace x Yorkshire x Duroc). Frequency of the AA genotype indel was at the highest level (66.67%). Frequency of A allele (0.81) was higher than that of b allele (0.19). This population followed Hardy-Weinberg equilibrium. Carcass weights and a^*^ values of the three genotypes were all significantly different (p < 0.05), respectively. However, backfat thickness, L^*^, b^*^, visual color, and pH of the three genotypes were not significantly different (p > 0.05). Visual color was negatively correlated with L^*^ (r = -0.521) and b^*^ (r = -0.390) values, L^*^ value was correlated with b^*^ (r = 0.419) value, and a^*^ value was positively correlated with b^*^ (r = 0.612) value. These results indicate that the *POU1F1* gene affected carcass weight and meat redness.

## Background

As of 2012, the annual average per capita meat consumption in Korea reached 40.6 kg, with pork accounting for the highest average consumption at 19.0 kg [1]. The Food and Agriculture Organization (FAO) revealed that global consumption of pork underwent an annual increase of 2.1% from 1999 to 2010 [2]. Due to an increase in average income, consumers have developed a preference for high quality pork, and research has been conducted to select genetically superior traits in pork [3]. In Korea, a crossbreed of three pig breeds, Landrace, Yorkshire, and Duroc (LY × D), has the largest market share based on its higher number of offspring, faster growth, and higher proportion of meat [4, 5] compared to other crossbreeds.

The *POU1F1* (POU domain, class 1, transcription factor 1, *Pit1*) gene is expressed in the pituitary gland of pigs. It is associated with growth regulation as well as secretion of growth hormones, prolactin, and thyroprotein β-subunit [6, 7]. *POU1F1* is located on chromosome 13 and consists of six exons and five introns, and it has been shown to influence quantitative traits related to pig growth [market weight, growth rate, and average daily gain (ADG)] as well as carcass traits (carcass weight, backfat thickness, amount of meat) [8–12]. Song et al. [13] reported that genetic change in intron 1 (insertion or deletion of 313 bp) of the *POU1F1* gene is related to pig growth, and the genotype frequencies of intron 1 vary among breeds. Their analysis of 15 breeds, including 11 breeds of Chinese traditional varieties and four traditional breeds, confirmed 100% frequency of the BB genotype in Chinese traditional varieties of Tibetan, Lingao, Rongchang, Songliao Black, and Min. Frequencies of the BB genotype were high in the Meishan, Erhualian, Fenjing, and Leping Spotted breeds, although the highest genotypes were found to be AA type, AB type, and BB type in the Landrace, Pietrain, and Duroc breeds, respectively. Further, A allele was shown to be present at higher frequencies than B allele in all four breeds [13]. Previous studies have reported the relationship between *POU1F1* gene intron region 3 and meat quality characteristics of the carcass [14] as well as that between daily gain and backfat thickness for the Landrace breed according to the *POU1F1* genotype [15]. However, further accurate research is required on *POU1F1* gene intron region 1 in crossbreeds of the three varieties.

Therefore, this study estimated *POU1F1* genotype frequency in 168 crossbred pigs that were raised domestically in order to obtain basic data on meat quality assessment for the establishment of a high-quality pork production system by analyzing meat color and carcass characteristics.

## Methods

### Test animals

Standard laboratory animals used in this study included 168 crossbred pigs, with a live weight of approximately 110 ± 5 kg. The breeds were Landrace, Yorkshire, and Duroc (LYD or YLD) and were raised in Gyeonggi-do, Chungcheong-do, and Jeolla-do on Nong-Hyup animal feed, which meets the specifications of the National Research Council (NRC).

### Sampling and genomic DNA extraction

To determine carcass grades and extract DNA, we transported animals to a slaughterhouse in Chungnam. After securing loin portions as laboratory samples, carcass grades were surveyed, and the meat was frozen and transported to the laboratory. The muscle tissues were then thawed at room temperature (-25°C), and an appropriate amount was purified using a QIAamp Mini kit (QIAGEN®, USA) following the manufacturer’s protocol. Purified genomic DNA was dissolved in distilled water or TE buffer (Tris-EDTA, pH 8.0) and collected by centrifugation. The amount of genomic DNA was confirmed by performing electrophoresis, followed by storage at -25°C until polymerase chain reaction (PCR) analysis.

### Genetic analysis

The primers used for amplification of the *POU1F1* gene are shown in Table 1. The primers used in the study were synthesized by the laboratory that amplified the intron 1 region of the *POU1F1* gene. For the normal genotype (AA type), an amplified product of approximately 1,091 bp in size was obtained, whereas BB type amplified product was 778 bp. The synthesized primers were diluted to a final concentration of 10 pmol.

**Table 1:** **Nucleotide sequences of primer pairs for PCR amplification of** ***POU1F1*** **DNA fragment**

Name of primer	Sequence	Size of products
POU1F1 gene in intron 1 region		1091 or 778 bp
Forward 5′-CAT TCC CAT TCT GCC ATT TG-3′ (20 mer)		
Reverse 5′-CCT GTT GCT GTG TTT CCC AG-3′ (22 mer)		

For the PCR mixture, prime Taq DNA polymerase from GeNet Biosystem (GeNet Bio. Co., Korea) was used. Each PCR mixture was 20 μL and consisted of 1× buffer (0.01 M Tris–HCl, 0.05 M KCl, 0.08% nonidet), 1.5 mM MgCl_2_, 1 mM of each dNTP, 10 pM of primers, and 2.5 U of Taq. PCR conditions were as follows: pre-denaturation at 94°C for 5 min, followed by 35 cycles of denaturation for 30 s at 94°C, annealing for 1 min at 55°C, and extension for 1 min at 72°C. PCR was completed with a final extension for 5 min at 72°C. The gene was amplified and analyzed using 1.5% agarose gel electrophoresis with TAE (40 mM Tris-acetate, 1.0 mM EDTA) buffer. The quality of DNA was checked by adding 0.1 mg of ethidium bromide to 1 μL of electrophoresis buffer in the gel and the electrophoresis medium, followed by electrophoresis for approximately 20 min at 100 V. A 100-bp ladder plus (MBI Fermentas Inc., USA) size marker was used as reference for the electrophoretic analysis, and images were obtained under UV illumination.

### Carcass trait according to genotype

Backfat thickness, carcass weight, meat color, and pH were surveyed as carcass characteristics. For backfat thickness, grading data from the slaughterhouse were used, whereas weight after slaughtering was measured and used as the carcass weight of each pork sample (live weight, 110 ± 5 kg). For meat color, Hunter L^*^ (lightness), a^*^ (redness), and b^*^ (yellowness) values on the cut surface of the fillet were measured using a color meter (Model NF333; Nippon Denshoku Co., Japan), and the standard meat color board at the time was Y = 92.40, X = 0.3136, and y = 0.3196. The visual color based on the meat color reference standard suggested by the National Pork Producers Council (NPPC) was directly compared for assessment based on seven steps, ranging from a light color score of 1 to a dark color score of 7. The pH of muscle was measured using a glass electrode pH meter (340; Mettler Toledo, Switzerland) after mixing 5 g of sample with 20 mL of distilled water, followed by homogenization for 1 min at 8,000 rpm using a homogenizer (Nissei, Model AM-7; Japan).

### Statistical analysis

The gene and genotypes of *POU1F1* intron 1 were analyzed using the SAS package (Statistical Analysis System, version 9.3). For intergroup genetic equilibrium, significance was tested using the χ^2^-test. After pigs were slaughtered, backfat thickness (mm), carcass weight (kg), meat color, and pH were determined to calculate the mean and standard deviations of each genotype, and correlation among carcass characteristics was calculated. For comparison of differences among groups, significance was tested at a 5% level by using Duncan’s multiple-range tests.

## Results and discussion

### *POU1F1*Intron 1 genetic analysis

**Figure 1:**
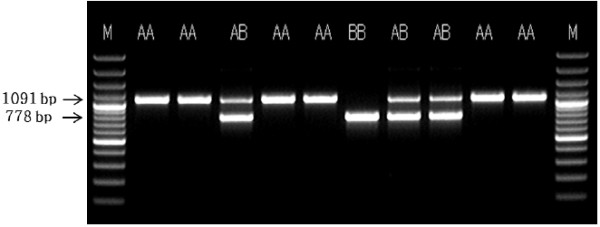
**PCR products of** ***POU1F1*** **gene intron 1 in pig samples.** M: Molecular size standard (100-bp DNA ladder plus), lanes 2–11: PCR products. AA genotype: 1091 bp; AB genotype: 1091 bp and 778 bp; BB genotype: 778 bp.

The results of PCR analysis of *POU1F1* intron 1 are shown in Figure 1. The PCR-amplified products consisted of three band types, namely AA and BB types as homozygotes and AB type as a heterozygote. Among these, AA type appeared as a single band of approximately 1091 bp, AB type appeared as two bands of 1091 and 778 bp, and BB type was a single band of 778 bp. These results are identical to those reported by Song et al. [13], who confirmed the presence of AA, AB, and BB types in intron 1. On the other hand, a previous study identified AA, AG, and GG types in the *FOU1F1* genotype of Korean beef [16].

The results of the analysis of the *POU1F1* intron 1 SNP genotype and allele frequencies are shown in Table 2. The genotypes were categorized as homozygotes, namely AA and BB types, as well as heterozygotes, which is AB type, along with the existence of alleles A and B. Of the 168 carcasses investigated, 112 were AA type, accounting for the highest percentage (66.67%); 48 (28.57%) and 8 (4.76%) were AA and BB types, respectively. These results are similar to those of Song et al. [13], who showed that the Landrace breed is AA type (0.76). On the other hand, they contradict the genotype frequencies of the Yorkshire breed, which was shown to be AB type (0.58), and Duroc breed, which was shown to be AA type (0.37). The discrepancies in these findings might be attributed to variations in gene distribution between crossbreeds and pure breeds, as crossbreeding involves changes in genotype frequencies. In contrast, the results of this study are similar to those of Franco et al. [15] and Yu et al. [17], who reported that the frequency of AB type is higher than that of AA type. Estimation of genotype frequencies revealed that A allele had a very high expression frequency (0.81), whereas B allele had a very low frequency (0.19). Meanwhile, Franco et al. [15] reported expression frequencies of 0.88 and 0.12 for A and B types in the Landrace breed, respectively. On the other hand, Yu et al. [17] and Song et al. [13] demonstrated high expression frequencies for A allele in the Yorkshire, Duroc, Landrace, and Hampshire breeds, whereas the frequency of B allele was high in the Chinese Meishan breed. These results might be due to differences among each breed. The results of conformity determined on the basis of Hardy–Weinberg law were not significant (p > 0.05), and this pork group was shown to maintain genetic equilibrium.

**Table 2:** **Genotypic frequencies of** ***POU1F1*** **gene and statistical test for Hardy-Weinberg equilibrium**

Genotype	No. of pigs	Percentage (%)	Gene frequency		Statistical test
			A	B	
AA	112 (110)	66.67	0.810	0.190	χ^2^-value : 0.908^NS^df = 1
AB	48 (52)	28.57			
BB	8 (6)	4.76			
Total	168 (168)	100	1		-

^NS^: Not-significant (p > 0.05).

( ): No. of expected.

### Carcass characteristics according to *POU1F1*Intron 1 genotype

The results of the analysis of backfat thickness, carcass weight, Hunter L^*^, a^*^, and b^*^values, meat color (visual color), and pH, which are the main carcass characteristics associated with the *POU1F1* SNP genotypes AA, AB, and BB, are shown in Table 3. The AA, AB, and BB genotype groups did not show intergroup significance, with an average backfat thickness of 12.14–13.33 mm (p > 0.05). These results are similar to those of Yu et al. [17], who confirmed lack of significant differences among genetic groups for backfat thickness. For carcass weight, the AA genotype group showed a significantly lower weight (78.76 kg) compared to the AB (92.00 kg) and BB (92.33 kg) types (p < 0.05). These results are similar to those of Yu et al. [17], who reported that carcass weight is greater in BB type than in AA type and suggested that carcass weight can be influenced by B allele.

**Table 3:** **Association analysis among genotype of** ***POU1F1*** **gene and carcass characteristics**

Trait	Genotype				Significant F-value
	AA	AB	BB	Mean ± SD	
Back fat Thickness (mm)	13.09 ± 0.92	12.14 ± 1.92	13.33 ± 1.86	12.59 ± 0.77	0.107 ^NS^
Carcass weight (kg)	78.76 ± 1.98^b^	92.00 ± 7.61^a^	92.33 ± 14.68^a^	81.86 ± 2.26	3.474^*^
*L* ^*^	39.54 ± 0.91	37.94 ± 1.78	38.11 ± 3.56	39.17 ± 0.79	0.343 ^NS^
*a* ^*^	17.42 ± 0.53^ab^	18.36 ± 1.73^a^	14.04 ± 3.55^b^	17.30 ± 0.52	3.258^*^
*b* ^*^	10.87 ± 0.40	10.90 ± 0.91	9.78 ± 1.92	10.79 ± 0.36	0.327 ^NS^
pH	5.70 ± 0.62	5.67 ± 0.13	5.59 ± 0.05	5.69 ± 0.05	0.195 ^NS^
Visual color^1)^	4.05 ± 0.17	4.63 ± 0.42	5.00 ± 0.07	4.22 ± 0.16	2.102 ^NS^

^1)^: 1 = pale, 7 = dark purple red.

^a, b^: Means with different superscripts in the same column differ significantly (p < 0.05).

*: p < 0.05, ^NS^: Not-significant.

No significant differences were observed in terms of meat color based on Hunter L^*^ (lightness) and b^*^ (yellowness) values, which were within the ranges of 37.94–39.54 and 9.78–10.90, respectively (p > 0.05). However, the *POU1F1* intron 1 gene was found to affect color expression since Hunter a* value (redness) was the highest in AB type pork (p > 0.05). In the meat color (visual color) assessment using the standard meat color board, the AA, AB, and BB genotypes showed values of 4.05, 4.63, and 5.00, respectively, with BB type showing a higher level of redness than AA type. In terms of pH, average frequency was 5.69 for the AB and BB genotypes, although no significant differences were observed between them. This result is similar to the findings of Yu et al. [17], who reported no differences among the three genotypes in terms of meat color.

In terms of average carcass characteristics, Oh et al. [18] obtained different results, with an average backfat thickness of 27 mm. Similarly, Choi et al. [19] reported relatively low Hunter L^*^ value and relatively high a^*^ and b^*^ values in the crossbreeds. Results for pH values were within the range of 5.75–5.49, which is almost the same as that reported by Kim et al. [20] and Oh et al. [18], whereas Choi et al. [19] reported slightly higher pH values. These differences might be attributed to different pig raising methods such as feeding.

### Correlation between carcass characteristics

The results of the correlation analysis of carcass characteristics are shown in Table 4. Meat color (visual color) showed a significant negative correlation with L^*^ (r = -0.52, p < 0.001) and b^*^ (r = -0.39, p < 0.01) values, whereas a low positive correlation was observed for backfat thickness, meat color (r = 0.12), and redness (r = 0.23). The L^*^ and b^*^ (r = 0.42, p < 0.05) values showed a low positive (+) correlation, whereas a^*^ and b^*^ (r = 0.61, p < 0.001) values showed a very high positive correlation.

**Table 4:** **Correlation coefficients among carcass characteristics in pigs**

Items	x _1_	x _2_	x _3_	x _4_	x _5_	x _6_	x _7_
Back fat Thickness (x_1_)	-	0.069	0.095	0.125	-0.174	0.229	0.066
Carcass weight (x_2_)		-	0.101	0.099	-0.216	-0.090	-0.0281
pH (x_3_)			-	0.135	-0.109	0.110	-0.097
Visual color (x_4_)				-	-0.521^***^	0.003	-0.390^**^
*L* ^*^ (x_5_)					-	-0.021	0.419^*^
*a* ^*^ (x_6_)						-	0.612^***^
*b* ^*^ (x_7_)							-

*: p < 0.05, **: p < 0.01, ***: p < 0.001.

These results are similar to those of Kim et al. [21], who reported that color and yellowness as well as redness and yellowness show high correlations, whereas color and redness show a negative correlation. The results on correlations among meat color (visual color), color (L^*^ value), redness (a^*^ value), and yellow (b^*^) values were very similar to those reported previously [22].

The results of this study suggest that changes in the *POU1F1* intron region 1 are related to carcass weight, meat color, as well as growth. Further extensive study on the relationship between pig growth and changes in *POU1F1* gene expression in relation to carcass characteristics is necessary in order to identify quantitative traits that might possibly be related to pig growth or meat quality of pork.

## Conclusion

The results of the analyses of carcass characteristics according to *POU1F1* genotypes of 168 domestically bred pig carcasses are as follows. Analysis of *POU1F1* intron 1 genotypes showed that the AA genotype has the highest frequency (66.7%), whereas frequencies of the AB and BB genotypes were 28.6% and 4.76%, respectively. The A allele showed a very high expression frequency (0.81), whereas B allele was estimated to have a low frequency (0.19). A χ^2^-test demonstrated that this allelic frequency was not in Hardy-Weinberg equilibrium. Backfat thickness and pH were not significantly different according to the genotype, but carcass weight was significantly higher in AB (92.00 kg) and BB (92.33 kg) types than in AA type (78.76 kg; p < 0.05). Further, Hunter a^*^ value was higher in AB type than in BB type (p < 0.05). In addition, meat color (visual color) showed a negative correlation with L^*^ (lightness) and b^*^ (yellowness) values. The results of this study showed that changes in the *POU1F1* gene influence carcass weight, backfat thickness, and meat color. Therefore, extensive investigation of the relationship between carcass characteristics and changes in the *POU1F1* gene is necessary to provide a reliable index for meat quality improvement.
